# The role of statins in the regulation of breast and colorectal cancer and future directions

**DOI:** 10.3389/fphar.2025.1578345

**Published:** 2025-05-14

**Authors:** Yexing Dang, Yu Zhang, Zhihao Wang

**Affiliations:** Department of Geriatrics, Jilin Geriatrics Clinical Research Center, The First Hospital of Jilin University, Changchun, China

**Keywords:** statins, breast cancer, colorectal cancer, anticancer mechanism, combination therapy

## Abstract

Statins, widely recognized as a cornerstone in the prevention of cardiovascular diseases, have garnered increasing attention in oncology due to their pleiotropic effects, particularly their potential roles in regulating breast and colorectal cancer. Emerging evidence suggests that statins may exert anticancer effects through multiple mechanisms, including the mitochondrial apoptosis pathway, the LKB1-AMPK-p38MAPK-p53-survivin signaling cascade, inhibition of the mevalonate pathway, modulation of the EGFR/RhoA and IGF-1 signaling pathways, and regulation of the BMP/SMAD4 signaling pathway. However, significant heterogeneity exists in the reported anticancer effects of statins, likely due to variations in statin type (lipophilic vs hydrophilic), dosage, treatment duration, and population-specific characteristics. These factors contribute to inconsistencies in study outcomes. Additionally, while combination therapies incorporating statins with chemotherapy and immunotherapy have demonstrated synergistic effects in certain studies, their clinical utility remains to be fully established. Nevertheless, current evidence suggests that statins may have a potential role in reducing breast and colorectal cancer-related mortality. Future research should prioritize elucidating their precise molecular mechanisms, defining dose–response relationships, developing personalized treatment strategies within the framework of precision medicine, and validating their efficacy through large-scale, long-term prospective studies. These efforts will provide a more robust scientific foundation for the clinical application of statins in oncology. This review systematically explores the role of statins in breast and colorectal cancer regulation, covering clinical evidence, underlying biological mechanisms, pharmacological distinctions, synergistic therapeutic potential, and translational medicine prospects.

## 1 Introduction

Cardiovascular diseases and cancer pose a dual global public health challenge, necessitating optimized prevention and treatment strategies. As a cornerstone in cardiovascular disease prevention, statins have attracted growing interest in oncology due to their pleiotropic effects, particularly their potential roles in regulating breast and colorectal cancer. Evidence suggests that statins may influence cancer biology through various mechanisms, including anti-inflammatory effects, inhibition of cell proliferation, and modulation of cancer-related cellular processes (Grabarek et al., 2021; Jiang et al., 2021; Morofuji et al., 2022; Bil, 2023; Chalhoub et al., 2023). However, the impact of statins on breast and colorectal cancer remains controversial, with studies reporting both anticancer potential and contradictory findings (Morofuji et al., 2022).

**TABLE 1:** A research summary on the prevention, treatment and combined therapy of breast and colorectal cancer with statins.

Statins	Disease	Combination agent	Forms	Dose	Findings	References
Atorvastatin	Breast cancer		*In vitro*	0–80 μM	Atorvastatin regulates the mitochondrial apoptosis pathway by modulating the Bax/Bcl-2 ratio and exerts antiproliferative effects by mediating cell death in MCF-7 cells through a synergistic effect with autophagy	Alarcon Martinez et al. (2018)
Lovastatin	Breast cancer		*In vitro*	0–50 μM	Lovastatin activates LKB1-AMPK-p38MAPK-p53-survivin cascade to cause MCF-7 cell death	Huang et al. (2020)
Mevastatin	Breast cancer		*In vitro* and vivo	0-16 μM (*In vitro*); 10 mg/Kg (*In vivo*)	Mevastatin enhances the anticancer effect of the histone deacetylase inhibitor LBH589 in triple-negative breast cancer cells by blocking the mevalonate pathway, which inhibits autolysosome maturation and thereby potentiates LBH589-induced apoptosis	Lin et al. (2017)
Atorvastatin, Pravastatin and Simvastatin	Breast cancer		A cohort study included New Zealand women first diagnosed with primary breast cancer between 2007 and 2016. Cases that could not be linked to pharmaceutical data or had died before their recorded date of breast cancer diagnosis were excluded. The final cohort comprised a total of 14,976 women	_	Postoperative statin use is associated with a 26% reduction in breast cancer-specific mortality, with more pronounced effects observed in patients with estrogen receptor-positive (ER+), postmenopausal, and advanced-stage disease	Scott et al. (2023)
_	Breast cancer		A cohort of 17,880 breast cancer patients, newly diagnosed between 1998 and 2009, was identified from English cancer registries	_	Statin use after a diagnosis of breast cancer had reduced mortality due to breast cancer and all causes	Cardwell et al. (2015)
Simvastatin	Breast cancer		A single-arm study enrolled 24 women with stage 0-II invasive breast cancer who were administered daily simvastatin (20 mg) for 2–4 weeks between diagnosis and surgical resection	20 mg/day	Simvastatin exerts its anti-tumor effects by inhibiting HMG-CoA reductase and blocking the mevalonate pathway, thereby affecting the activity of Ras and RhoGTAases. It may promote cell cycle arrest and apoptosis, thus playing an anti-tumor role	Kamal et al. (2024)
Simvastatin	Breast cancer	Fluorouracil, adriamycin and cyclophosphamide (FAC)	A randomized, double-blinded, placebo-controlled trial in two centers of Indonesia:patients were randomly assigned to FAC plus simvastatin (40 mg/day orally) or FAC plus placebo (40 mg/day) for 21 days. The FAC regimen was repeated every 3 weeks	40 mg/day	Simvastatin enhances doxorubicin-induced apoptosis and cell cycle arrest (G1/S phase) by inhibiting the mevalonate-Rho/ROCK signaling pathway and may overcome chemotherapy resistance by modulating the ABCB1 transmembrane protein, thereby synergistically improving treatment outcomes	Yulian et al. (2021)
Pitavastatin and simvastatin	Breast cancer	Doxorubicin/Cyclophosphamide	*In vitro*	Pitavastatin: 0-50 μM and simvastatin: 0-25 μM	Combined therapy upregulate the pro-apoptotic gene Bax and downregulate Bcl‐2 expression, enhancing the cytotoxic effects of chemotherapy on MDA-MB-231 and MCF7 cells	Dewidar et al. (2023)
Atorvastatin and simvastatin	Breast cancer	OSI-906, doxorubicin and docetaxel	*In vitro*	Atorvastatin:0.4–61 μM and simvastatin:0.2–50 μM	Atorvastatin and simvastatin exhibit stronger proliferation inhibition and apoptosis induction in triple-negative breast cancer (TNBC) cells, particularly those harboring p53 mutations. Their combination with IGF-1R inhibitors or chemotherapeutic agents such as doxorubicin further enhances therapeutic efficacy	O’Grady et al. (2022)
Lovastatin	Breast cancer	Paclitaxel	*In vitro* and vivo	0-20 μM (*In vitro*); 50 mg/Kg (*In vivo*)	Lovastatin can inhibit paclitaxel-induced PD-L1 expression and enhance CD8^+^ T cell cytotoxicity, thereby improving breast cancer prognosis	Li et al. (2024)
Atorvastatin	Colorectal cancer		*In vivo*	0%–0.04% (w/w)	Atorvastatin induces G0/G1 phase cell cycle arrest in colorectal cancer cells, activates apoptosis-related proteins, suppresses inflammatory cytokines (IL-1β, IL-6, TNF-α), and downregulates the EGFR/RhoA signaling pathway, thereby exerting anticancer effects	Wu et al. (2017)
Simvastatin	Colorectal cancer		*In vitro*	2.5-20 μM	Simvastatin significantly reduced the expression of IGF-1R and inhibited the activation of the ERK and Akt signaling pathways induced by IGF-1	Jang et al. (2016)
_	Colorectal cancer		A cohort of 69,272 statin users and 94,753 controls from two registries unique to the Netherlands	_	Statins significantly reduced the risk of colorectal cancer with high SMAD4 expression (OR 0.64) by activating the BMP/SMAD4 signaling pathway	Ouahoud et al. (2022)
_	Colorectal cancer		A cohort study: 10,743 patients with Stage I-III rectal cancer who underwent curative surgery, of whom 26% (2,797 individuals) were classified as having ongoing statin therapy. Data for these patients were extracted from the Swedish Colorectal Cancer Register	_	Continuous statin use was significantly associated with reduced all-cause mortality (HR 0.66, 95% CI 0.60–0.73) and cancer-specific mortality (HR 0.60, 95% CI 0.47–0.75) for up to 5 years post-surgery	Pourlotfi et al. (2021)
_	Colorectal cancer		A meta-analysis included 5 retrospective case-control studies (including 475 statins users and 1,925 no-statin users) and 11 prospective cohort studies (including 40,659 statins users and 344,459 no-statin users)	_	Statin use has been strongly correlated with decreased overall and cancer-specific mortality in colorectal cancer	Li et al. (2021)
Rosuvastatin	Colorectal cancer	Regorafenib	*In vitro* and vivo	1.25-200 μM (*In vitro*); 50 mg/Kg (*In vivo*)	Rosuvastatin and regorafenib synergistically inhibit MEK/ERK phosphorylation and enhance pro-apoptotic effects	Yuan et al. (2023)
Simvastatin	Colorectal cancer	Oxaliplatin	*In vitro* and vivo	0-10 μM (*In vitro*); 20 mg/Kg (*In vivo*)	In KRAS-mutated colorectal tumors, simvastatin can inhibit KRAS prenylation and activate endoplasmic reticulum stress, thereby increasing tumor immunogenicity. When combined with chemotherapeutic agents such as oxaliplatin, they significantly boost antitumor immune responses	Nam et al. (2021)

While some studies indicate that statins may significantly reduce cancer-related mortality in patients with breast and colorectal cancer, substantial heterogeneity persists across research findings (Chen et al., 2022; Liang et al., 2023; Zhou et al., 2023). Variability in statin type (lipophilic vs hydrophilic), dosage, duration of use, and population-specific characteristics may influence therapeutic outcomes, contributing to these inconsistencies. Additionally, although combination therapies incorporating statins with chemotherapy and immunotherapy have demonstrated synergistic effects in certain studies, their clinical utility remains to be fully elucidated.

Given the limitations of current research, future studies should prioritize large-scale cohort investigations with extended follow-up periods to clarify the dose–response and time–response relationships between statin use and cancer outcomes. Further exploration of the molecular mechanisms underlying statins’ effects on cancer, including their roles in tumor metabolic reprogramming, key signaling pathways, and modulation of the tumor immune microenvironment, is essential. Moreover, the development of personalized treatment strategies, accounting for differences in statin isoforms and population-specific factors, is crucial for optimizing their therapeutic potential (Zaleska et al., 2018; Saka-Herrán et al., 2022; Stepien et al., 2022).

**FIGURE 1:**
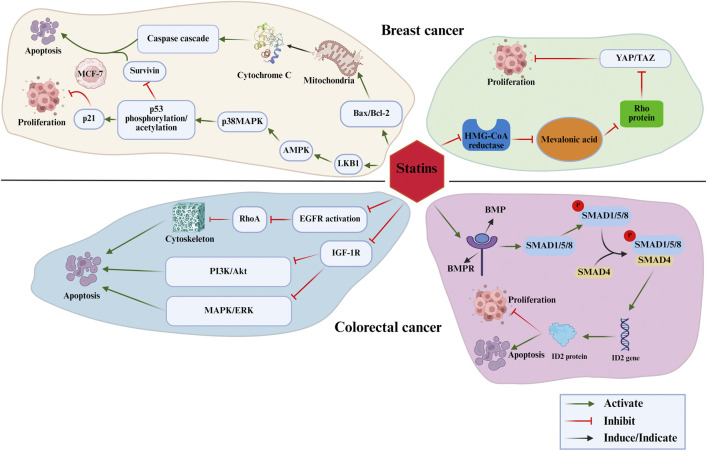
The mechanisms by which statins influence breast and colorectal cancer.

This review aims to systematically examine the role of statins in breast and colorectal cancer regulation from multiple perspectives, including clinical evidence, underlying biological mechanisms, pharmacological distinctions, synergistic therapeutic potential, and translational medicine prospects. By providing a comprehensive synthesis of current knowledge, this review seeks to outline a roadmap for future research aimed at harnessing the anticancer properties of statins. A deeper understanding of their pleiotropic effects is essential for advancing novel therapeutic strategies. Figure 1 presents an overview of the proposed mechanisms by which statins influence breast and colorectal cancer.

## 2 Existing evidence on the anticancer effects of statins in breast and colorectal cancer

### 2.1 Research progress and molecular mechanisms of statins in the prevention and treatment of breast cancer

Statins have demonstrated potential in the prevention and treatment of breast cancer. A meta-analysis has reported that statin use may reduce mortality in breast cancer patients (Liu et al., 2017). In a large cohort study involving 14,976 breast cancer patients, post-diagnostic statin use was associated with a significantly reduced risk of breast cancer-specific mortality (adjusted HR 0.74, 95% CI 0.63–0.86), with more pronounced protective effects observed in estrogen receptor-positive (ER+) patients, postmenopausal women, and those with advanced-stage disease (Scott et al., 2023). Similarly, post-diagnosis statin use has been linked to reduced breast cancer-specific and all-cause mortality (Cardwell et al., 2015). Beyond these epidemiological findings, statins may exert anticancer effects through multiple molecular mechanisms, including the inhibition of cancer stem cell activity and modulation of the tumor microenvironment. The primary mechanisms underlying these effects are as follows: 1) Mitochondrial Apoptosis Pathway: Atorvastatin regulates the mitochondrial apoptosis pathway by modulating the Bax/Bcl-2 ratio, thereby promoting autophagy-mediated MCF-7 cell death and exerting antiproliferative effects (Alarcon Martinez et al., 2018). 2) LKB1-AMPK-p38MAPK-p53-Survivin Signaling Cascade: Lovastatin activates the LKB1-AMPK-p38MAPK signaling cascade, leading to the phosphorylation and acetylation of p53. This process subsequently suppresses survivin expression while upregulating p21, resulting in cell cycle arrest and apoptosis, which effectively inhibits MCF-7 cell proliferation (Huang et al., 2020). 3) Inhibition of the Mevalonate Pathway: Statins suppress the mevalonate pathway, thereby reducing tumor cell proliferation, inducing apoptosis, and enhancing the tumor microenvironment, which in turn improves the efficacy of targeted therapies (Hyder et al., 2021; Xie et al., 2021; Kamal et al., 2024; Karimi Jirandehi et al., 2024; Tang et al., 2024). Specifically, statins inhibit the YAP/TAZ signaling pathway within the mevalonate pathway, thereby disrupting cancer cell proliferation and survival. By preventing the prenylation and membrane localization of Rho proteins, statins promote the phosphorylation, degradation, and impaired nuclear translocation of YAP/TAZ, leading to reduced transcriptional activity and suppression of breast cancer (Hwang et al., 2019). Additionally, mevastatin has been shown to inhibit autophagosome maturation by targeting the mevalonate pathway, thereby enhancing LBH589-induced apoptosis in triple-negative breast cancer cells (Lin et al., 2017).

These studies collectively provide a strong theoretical foundation for further investigation into the potential applications of statins in breast cancer prevention and treatment.

### 2.2 Research progress and molecular mechanisms of statins in the prevention and treatment of colorectal cancer

Statins have shown considerable potential in the prevention and treatment of colorectal cancer. A study involving 10,743 patients who underwent surgery for rectal cancer found that continuous statin use was significantly associated with reduced all-cause mortality (HR 0.66, 95% CI 0.60–0.73) and cancer-specific mortality (HR 0.60, 95% CI 0.47–0.75) for up to 5 years post-surgery (Pourlotfi et al., 2021). Moreover, statin use has been strongly correlated with decreased overall and cancer-specific mortality in colorectal cancer (CRC) (Li et al., 2021). Notably, both pre- and post-diagnosis statin use has been linked to a significant reduction in cancer-specific mortality (Pourlotfi et al., 2021; Kim et al., 2022). The molecular mechanisms underlying these effects include: 1) Regulation of the EGFR/RhoA Signaling Pathway: Atorvastatin induces G0/G1 phase cell cycle arrest in colorectal cancer cells, activates apoptosis-related proteins, suppresses inflammatory cytokines (IL-1β, IL-6, TNF-α), and downregulates the EGFR/RhoA signaling pathway, thereby exerting anticancer effects (Wu et al., 2017). 2) Modulation of the IGF-1 Pathway: Simvastatin promotes apoptosis in colorectal cancer cells by inhibiting IGF-1-induced ERK and Akt expression and further enhancing apoptosis through the downregulation of IGF-1R (Jang et al., 2016). 3) BMP/SMAD4 Signaling Pathway: A cohort study demonstrated that statins significantly reduced the risk of colorectal cancer with high SMAD4 expression (OR 0.64) by activating the BMP/SMAD4 signaling pathway. However, their effects were not observed in tumors harboring KRAS/BRAF mutations, suggesting that statins’ anticancer activity is mediated through the SMAD4 pathway rather than the RAS/RAF pathway (Ouahoud et al., 2022). These findings underscore the anticancer potential of statins in colorectal cancer and highlight the necessity for further investigation into their molecular mechanisms. Table 1 presents the summary of research on statins for the prevention and treatment of breast and colorectal cancer.

## 3 Controversies Surrounding the association of statins with breast and colorectal cancer

### 3.1 Controversies surrounding statins in the prevention and treatment of breast cancer

While some studies suggest that statins may lower the risk of certain cancers, their definitive preventive role in breast cancer remains unconfirmed. This ongoing debate primarily stems from methodological limitations, as most studies are observational, lack randomization, and are prone to selection bias. Additionally, substantial heterogeneity exists among study populations, including variations in age distribution, cancer subtypes, and statin dosages. The controversy is particularly evident in breast cancer research. For example, Nowakowska et al. reported that initiating statin therapy within 12 months of diagnosis improved overall survival (OS) and breast cancer-specific survival (BCSS) in patients with triple-negative breast cancer (TNBC). However, this effect was not observed in non-TNBC subtypes, suggesting that molecular subtypes may influence treatment efficacy (Nowakowska et al., 2021). Similarly, a retrospective study of postmenopausal women with hormone receptor-positive early breast cancer indicated that the adverse impact of hydrophilic statins (pravastatin and rosuvastatin) on disease-free survival (DFS) diminished after adjusting for confounding factors. However, given the study’s limited sample size and retrospective design, these findings require further validation (Minichsdorfer et al., 2022). To establish a clearer understanding of statins’ anticancer effects, future research must address these limitations through well-designed randomized controlled trials and larger, more diverse study populations.

### 3.2 Controversies surrounding statins in the prevention and treatment of colorectal cancer

For colorectal cancer, studies have suggested that statins may reduce the risk of developing the disease; however, this effect remains controversial. The primary source of this debate lies in the inconsistent findings across different studies, with some failing to confirm a preventive role for statins in colorectal cancer. These discrepancies may stem from variations in study populations, differences in statin dosage and duration of use, and the physicochemical properties of statins (lipophilic vs hydrophilic). For instance, a real-world data analysis from South Korea found no statistically significant association between statin use and improved survival in colorectal cancer patients (Woo and Shin, 2024). Similarly, a meta-analysis indicated that statins may have chemopreventive potential in individuals without inflammatory bowel disease (IBD) but not in IBD patients, underscoring the impact of population heterogeneity on research outcomes (Sun et al., 2023). In contrast, a cohort study from Hong Kong reported that patients who used statins for more than 90 days had a lower incidence of post-colonoscopy colorectal cancer (PCCRC) within 5 years; however, these findings require validation through prospective studies (Cheung et al., 2019).

The controversy over the anticancer effects of statins is further reflected in differences between their lipophilic (lovastatin, simvastatin, fluvastatin, atorvastatin, pitavastatin) and hydrophilic (pravastatin, rosuvastatin) subtypes. Evidence suggests that statins with distinct physicochemical properties may exert anticancer effects through different mechanisms, contributing to the variability in study outcomes. Key factors influencing this debate include differences in sample selection criteria, diverse dosing regimens, and the failure of some studies to differentiate between the effects of lipophilic and hydrophilic statins. Additionally, inadequate adjustment for confounding variables—such as patients’ baseline disease status and cancer molecular subtypes—may further obscure the true impact of statins (Frisk et al., 2021; Kwon et al., 2021; Hou and Shao, 2022; Zhou et al., 2023).

The potential role of statins in the prevention and treatment of both breast and colorectal cancer remains unresolved. Future research should prioritize long-term cohort studies with rigorous methodologies to better evaluate the specific effects of statins on different cancer subtypes.

## 4 Combination of statins with chemotherapy and immunotherapy in breast and colorectal cancer

### 4.1 Combination use of statins in the treatment of breast cancer

In breast cancer therapy, statins have demonstrated significant synergistic effects, particularly when combined with chemotherapeutic agents. For example, simvastatin enhances doxorubicin-induced apoptosis and G1/S phase cell cycle arrest by inhibiting the mevalonate-Rho/ROCK signaling pathway. Additionally, it may overcome chemotherapy resistance by modulating the ABCB1 transmembrane protein, thereby improving treatment efficacy, especially in HER2-positive patients (Yulian et al., 2021). Similarly, atorvastatin indirectly suppresses cancer cell proliferation by inhibiting cholesterol synthesis and downregulating pathways such as RhoA/EGFR, leading to reduced expression of inflammatory, angiogenic, and metastatic factors. This effect is further enhanced when atorvastatin is combined with beta-blockers in breast cancer treatment (Phadke and Clamon, 2019). In combination therapies, simvastatin and pitavastatin have been shown to enhance the cytotoxic effects of doxorubicin and cyclophosphamide by upregulating the pro-apoptotic gene Bax and downregulating Bcl-2, thereby increasing chemotherapy-induced apoptosis in MDA-MB-231 and MCF7 breast cancer cells (Dewidar et al., 2023). Moreover, atorvastatin and simvastatin exhibit stronger proliferation inhibition and apoptosis induction in triple-negative breast cancer (TNBC) cells, particularly those harboring p53 mutations. Their combination with IGF-1R inhibitors or chemotherapeutic agents such as doxorubicin further enhances therapeutic efficacy, highlighting their potential in precision treatment for TNBC and the need to explore the predictive value of p53 status (O'Grady et al., 2022).

Emerging evidence also suggests that statins may play a role in breast cancer immunotherapy. The combination of statins and Th1 cytokines significantly enhances apoptosis and causes Ras proteins to relocate from the cell membrane in breast cancer cells. In a HER2-positive breast cancer mouse model, this approach, when combined with dendritic cell-based immunotherapy, markedly suppresses tumor growth, providing experimental support for statins as potential adjuncts in immunotherapy (Oechsle et al., 2020). Furthermore, Mendelian randomization analyses and *in vitro*/*in vivo* experiments have demonstrated that statins, such as lovastatin, can inhibit paclitaxel-induced PD-L1 expression and enhance CD8^+^ T cell cytotoxicity, thereby improving breast cancer prognosis. These findings suggest that statins may facilitate the transition of “cold” tumors to “hot” tumors, reinforcing their potential as adjuvants in breast cancer immunotherapy (Li et al., 2024). Collectively, these findings underscore the potential of statins as valuable adjuncts in breast cancer treatment, warranting further investigation into their role in combination therapies.

### 4.2 Combination use of statins in the treatment of colorectal cancer

In colorectal cancer therapy, statins exhibit significant synergistic effects, particularly when combined with chemotherapeutic agents. Their therapeutic action operates through two primary mechanisms: enhancing chemotherapy efficacy by reducing inflammation and inducing tumor cell apoptosis, and suppressing tumor metastasis by inhibiting angiogenesis. However, pharmacokinetic interactions—especially those involving the CYP450 enzyme system—require clinical attention when statins are co-administered with anticancer drugs. The combination of statins with oxaliplatin (L-OHP) has been shown to enhance antitumor efficacy in KRAS-mutated colorectal cancer cells while also mitigating L-OHP-induced neuropathy. Specifically, statins potentiate L-OHP’s anticancer effects and alleviate neuropathy by inhibiting ERK1/2 activation, providing experimental support for their potential role as adjuvants in colorectal cancer chemotherapy (Tsubaki et al., 2023). Additionally, the combination of regorafenib and rosuvastatin has been demonstrated to synergistically inhibit MAPK signaling both *in vitro* and *in vivo*, achieving enhanced antitumor efficacy in colorectal cancer (Yuan et al., 2023).

Beyond direct tumor inhibition, statins have been found to modulate the gut microbiota, notably enriching *Lactobacillus* reuteri and upregulating the metabolite indole-3-lactic acid (ILA), which collectively contribute to tumor suppression. This discovery opens new avenues for the application of statins in colorectal cancer treatment (Han et al., 2023).

Recent studies have also highlighted the immunomodulatory potential of statins in colorectal cancer. Lovastatin, when used to facilitate cGAS-STING activation via liposomal delivery, significantly enhances both chemotherapy and immunotherapy efficacy (Yang et al., 2024). Furthermore, statins enhance immune responses by inhibiting key tumor signaling pathways, thereby synergizing with immune checkpoint inhibitors, such as PD-1 inhibitors, to improve treatment outcomes (Cortellini et al., 2020). In KRAS-mutated colorectal tumors, statins have been shown to inhibit KRAS prenylation and activate endoplasmic reticulum stress, thereby increasing tumor immunogenicity. When combined with chemotherapeutic agents such as oxaliplatin, they significantly boost antitumor immune responses, offering a promising combinatorial strategy for colorectal cancer immunotherapy (Nam et al., 2021).

Given the growing body of evidence supporting the adjunctive role of statins in colorectal cancer therapy, rigorous clinical trials are warranted to further evaluate their efficacy and optimize their integration into treatment regimens. Table 1 presents the summary of research on statins for combination therapy in breast and colorectal cancer.

## 5 Future directions in the development of statins

The development of statins in cancer therapy is progressing toward precision and multifunctionality. Optimizing the molecular structure to enhance anticancer efficacy has become a prominent research direction. Tailoring statin molecules with specific physicochemical properties (lipophilic or hydrophilic) to target different cancer types can significantly improve drug permeability and targeting capabilities (Zaleska et al., 2018). Concurrently, novel drug delivery systems are being actively explored. Studies have shown that combining statins with chemotherapy and immunotherapy through nanotechnology can optimize drug release kinetics and bioavailability, thereby enhancing therapeutic outcomes (Heart Protection Study Collaborative, 2011; Malenda et al., 2012).

Innovatively, the development of dual-functional cardiovascular drugs, such as statins with both lipid-lowering and anticancer properties, has garnered significant attention (Zaleska et al., 2018; Grabarek et al., 2021; Morofuji et al., 2022; Chalhoub et al., 2023). However, while pursuing the anticancer effects of statins, it is essential to carefully balance their potential side effects on liver function and the immune system (Frisk et al., 2021; Grabarek et al., 2021; Kwon et al., 2021). Achieving this requires integrating the strengths of molecular biology, oncology, and cardiology, as well as leveraging precision medicine and big data analytics to optimize candidate molecules. Collaborating with oncology experts to design rigorous clinical trials is also crucial to clarify the anticancer potential of cardiovascular drugs and identify appropriate target populations.

At the molecular level, developing statins with high selectivity and potency for targeting specific cancer signaling pathways, such as the YAP/TAZ pathway, has become a key strategy (Taccioli et al., 2015; Iannelli et al., 2020). The use of nanodrug delivery systems can significantly enhance drug targeting in the tumor microenvironment while minimizing systemic toxicity (Mehibel et al., 2018). Furthermore, the combination of metabolic inhibitors with immune modulators presents a promising avenue for developing novel cardiovascular drugs targeting tumor metabolic pathways (Nielsen et al., 2012; Wang et al., 2016; Mei et al., 2017; Yang et al., 2020; Cantini et al., 2021; Majidi et al., 2021). However, the heterogeneity of cancer subtypes presents a significant challenge in drug development, requiring large-scale clinical trials and biomarker studies to overcome. Establishing interdisciplinary collaboration mechanisms—integrating medicinal chemistry, clinical oncology, and bioinformatics—will be pivotal in accelerating the drug development process. Additionally, combining systems biology with artificial intelligence offers powerful tools for efficiently screening and validating cardiovascular drugs with anticancer potential.

Dose optimization strategies based on population data are essential for maximizing the anticancer and cardiovascular protective effects of statins. Developing statins with higher tissue selectivity to reduce toxicity in normal tissues and improve clinical applicability is a key focus. The application of gene-editing technologies offers new opportunities to develop cardiovascular drugs targeting specific genetic mutations, potentially lowering individualized cancer risks. However, the safety and efficacy of these innovative therapies need validation through long-term follow-up and large-scale randomized controlled trials.

Establishing data-sharing platforms between the cardiovascular and cancer fields, along with utilizing big data and machine learning techniques to identify potential drug targets, will provide crucial support for future research. Finally, the creation of a multicenter collaboration framework will facilitate the connection between basic research and clinical trials, helping to accelerate the translational application of statins in cancer therapy. Developing advanced drug delivery systems and exploring personalized treatment strategies will be essential to fully harness the anticancer potential of statins.

## 6 Conclusion

Statins have shown promising potential in the regulation of breast and colorectal cancer, though their effects remain controversial. Existing evidence suggests that statins may exert anticancer effects through mechanisms such as inhibition of the mevalonate pathway and modulation of the tumor microenvironment. However, the variability in study results underscores the need for further investigation. Future research should focus on elucidating the underlying molecular mechanisms, optimizing dosing regimens, and validating the specific effects of statins in different cancer subtypes through large-scale clinical trials. The integration of interdisciplinary collaboration and precision medicine strategies will offer new opportunities for the translational application of statins in cancer prevention and treatment. Key research areas include: 1) Determining the molecular mechanisms by which statins influence cancer risk; 2) Establishing the dose–response relationship and identifying the most effective dosing regimens; 3) Designing personalized treatment options tailored to individual patients; 4) Developing and validating hypotheses related to the anticancer efficacy of statins in large, long-term studies; and 5) Exploring new drug delivery methods and combining statins with other anticancer treatments. Addressing these current controversies and optimizing statin use will be crucial for their successful application in cancer therapy.
